# Exploring Dermatomyositis through an Interdisciplinary Lens: Pearls from Dermatology and Rheumatology

**DOI:** 10.1016/j.ijwd.2021.09.007

**Published:** 2021-09-24

**Authors:** Bina Kassamali, Daniel R. Mazori, Avery H. LaChance, Lisa Christopher-Stine

**Affiliations:** a Department of Dermatology, Brigham and Women's Hospital, Boston, MA; b Harvard Medical School, Boston, MA; c Department of Medicine, Division of Rheumatology, Johns Hopkins University School of Medicine, Baltimore, MD

**Keywords:** dermatomyositis, anti-melanoma differentiation-associated gene 5 (anti-MDA5), antinuclear matrix protein 2 (anti-NXP2), dermatology, rheumatology, connective tissue disease, multidisciplinary care

 

**What is known about this subject in regard to women and their families?**

• Women are affected by DM two to three times more often than men (Marvi et al., 2012).
• In addition to the fact that women are more susceptible to autoimmune disease in general (Angum et al., 2020), research has shown sex differences in risk factors for dermatomyositis. There is evidence that ultraviolet radiation increases the risk of development of dermatomyositis in women, but not in men (Love et al., 2009).
• There is also evidence of sex differences in autoantibody profiles and cytokine expression (Hamaguchi et al., 2011). Specifically, gene expression levels of the adipokine, visfatin, are significantly elevated in patients with newly diagnosed DM, and are associated with female over male sex (Olazagasti et al., 2015).
• Dermatomyositis has shown to have a significant impact on quality of life with significantly worse mental health scores, when compared to various other dermatologic and non-dermatologic diseases (Goreshi et al., 2011).
• The most frequent dermatomyositis-associated malignancies for women are breast and ovarian cancer (Hsu et al., 2021).

**What is new from this article as messages for women and their families?**

• It is crucial to screen our female patients for potential underlying malignancy with a heightened focus on ovarian and breast cancer.
• Given that the prevalence of DM is vastly greater among women than men, more women are affected by low overall mood and psychosocial distress. This points to the importance for physicians to include psychosocial interventions for all patients with DM.
• When coming up with a management plan, it is important to ask women who are in childbearing years about their thoughts around family planning. Medications such as methotrexate (MTX) and mycophenolate mofetil (MMF) have teratogenic effects and are therefore unsafe for women who are planning families.
• An alternative, and effective, medication that is safe in pregnancy and breast feeding is IVIG.

## Introduction

Dermatomyositis (DM) is a rare autoimmune disease characterized by inflammatory and degenerative changes in the skin and muscles. Beyond this, DM may be associated with significant systemic complications and is paraneoplastic in up to 30% of patients (Wolff et al., 2017). Women are affected by DM two to three times more often than men (Marvi et al., 2012). There is evidence of sex differences in both autoantibody profiles and cytokine expression (Hamaguchi et al., 2011). Specifically, gene expression levels of the adipokine, visfatin, are significantly elevated in patients with newly diagnosed DM, and are associated with female over male sex (Olazagasti et al., 2015). The presentation and findings in DM may vary significantly from patient to patient, highlighting the value of collaboration across specialties to optimize patient care. Dermatologists and rheumatologists often work closely together to guide the diagnosis, work-up, and management of patients with DM. Herein, we present two patients with DM and discuss both dermatologic and rheumatologic pearls that are essential for the diagnosis and treatment of patients with this complex disease.

### Patient 1

**Fig. 1:**
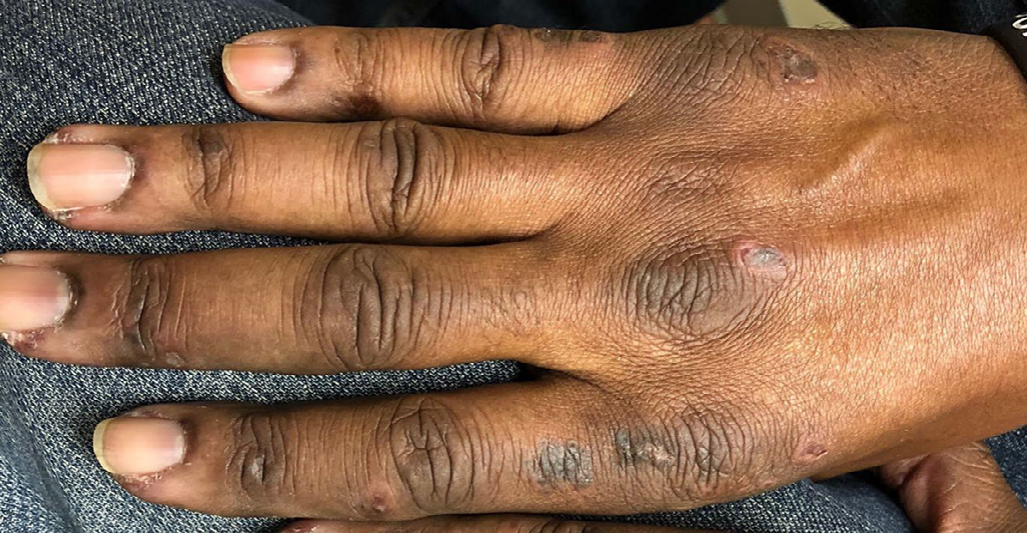
Gottron's papules with overlying ulcers are highly specific for MDA-5 dermatomyositis. Cutaneous features of dermatomyositis can appear more hyperpigmented than erythematous in patients with skin of color.

A 32 year-old woman presented with a six-month history of bilateral symmetric swelling of her knuckles associated with 1 hour of morning stiffness. Due to these symptoms, she had blood work that showed a positive rheumatoid factor (RF), positive anti-cyclic citrullinated peptide (anti-CCP) antibody, and mildly elevated erythrocyte sedimentation rate (ESR) and C-reactive protein (CRP). She became more alarmed when she noted bumps on her knuckles, some with overlying ulcers despite no history of trauma (Fig. 1).

Dermatology: The dorsal hands are a crucial window into the diagnosis of DM and can provide a multitude of clinical clues to support this diagnosis. Gottron's papules are one of the most characteristic findings of DM, manifesting as erythematous to violaceous papules and/or plaques with or without scale over the bilateral metacarpophalangeal (MCP), proximal interphalangeal (PIP), and/or distal interphalangeal (DIP) joints (Fig. 1). Occasionally, Gottron's papules can involve the sides of the fingers and the interphalangeal skin. Stretch is believed to play a role in the pathophysiology of Gottron's papules, which is why they can be more prominent on or isolated to a patient's dominant hand (Kurtzman et al., 2016).

**Fig. 2:**
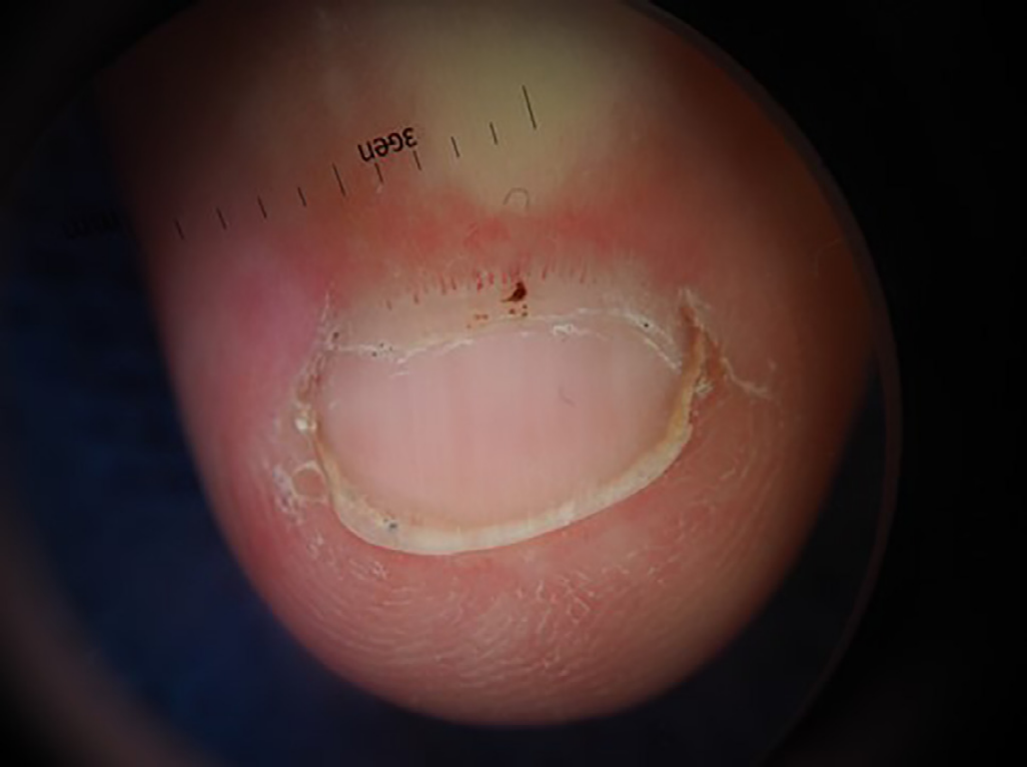
Dermoscopic image of nailfold capillary dilation alternating with dropout, focal hemorrhage, and cuticular dystrophy.

As there are several mimickers of Gottron's papules (e.g. verruca, knuckle pads, papular granuloma annulare), evaluating these lesions alongside the proximal nail folds and cuticles is key (Fig. 2). All patients with DM should have at least early proximal nailfold abnormalities (periungual erythema, nailfold capillary dilation alternating with dropout, and cuticular dystrophy). Seeing Gottron's papules concurrent with nailfold abnormalities is key to helping confirm the diagnosis of DM from the hands alone.

**Fig. 3:**
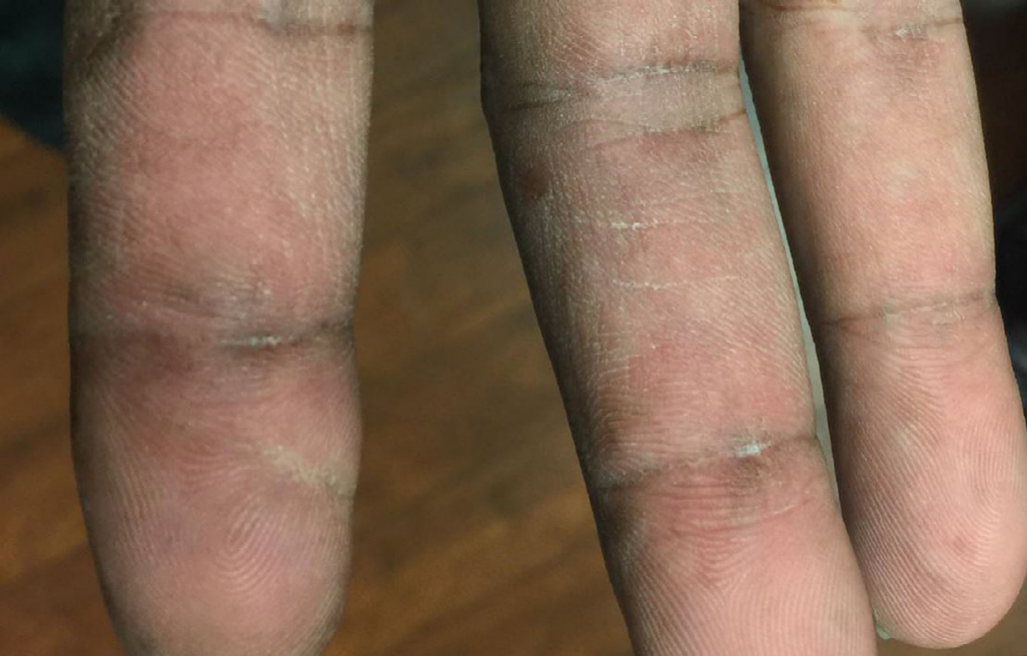
Painful palmar papules are highly specific for MDA-5 dermatomyositis.

**Fig. 4:**
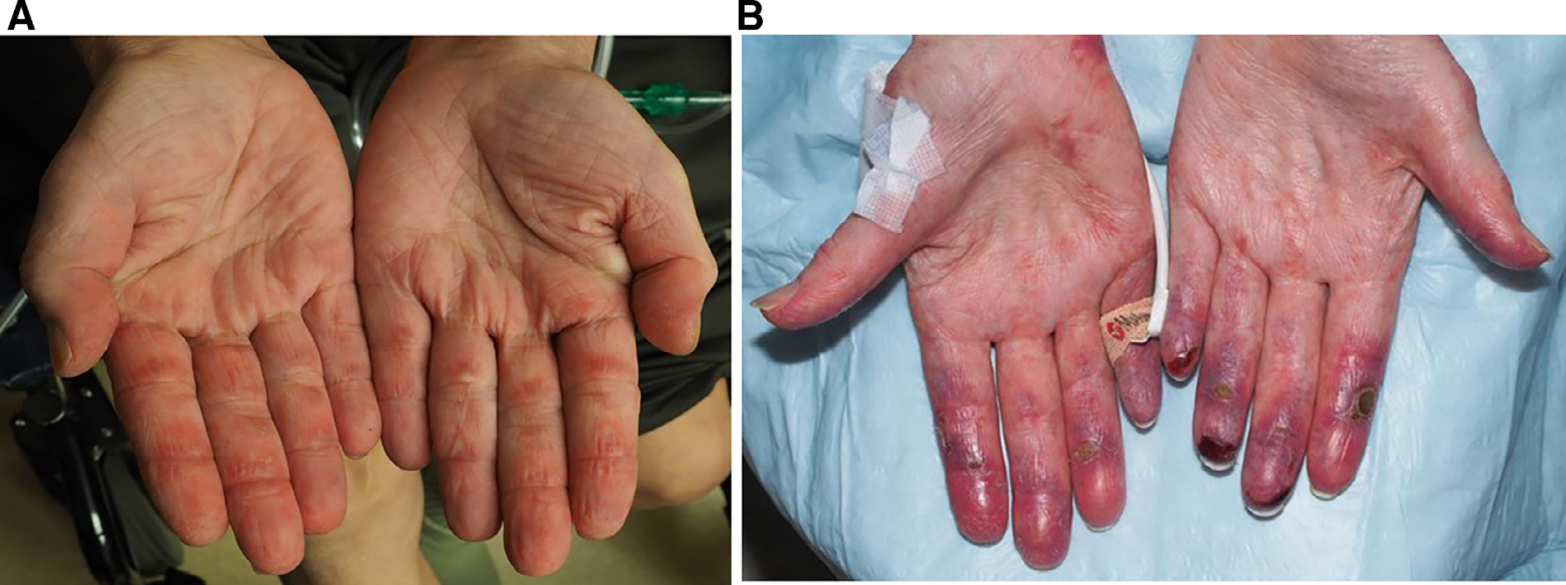
The spectrum of painful palmar macules in MDA-5 dermatomyositis from more mild (A) to severe/ulcerative (B).

Ulcers overlying Gottron's papules are highly concerning for a subtype of DM associated with the anti-melanoma differentiation-associated gene 5 (MDA5) autoantibody. These ulcers, along with painful palmar macules and papules (Fig. 3, Fig. 4), are highly specific for this phenotype, which confers a higher risk of interstitial lung disease (ILD) (Kurtzman and Vleugels, 2018). Because the autoantibodies for DM can take months to return, in almost all cases, DM should be considered a clinical diagnosis based on physical exam, with work-up and monitoring guided by the patient's findings. For this patient, her physical exam findings alone are highly suggestive of anti-MDA5 DM, prompting need for early and close screening for ILD.

**Rheumatology:** Bilateral hand swelling reminiscent of rheumatoid arthritis (RA) may be the initial feature of anti-MDA5 DM or another phenotype of DM called antisynthetase syndrome. In fact, some patients with DM will have a positive RF or anti-CCP antibody which could be mistaken for seropositive RA. Given that isolated inflammatory arthritis is often the initial presenting symptom of patients with inflammatory myopathy, it is important to look for dermatologic or systemic clues to help differentiate inflammatory myopathies from RA. Often, DM is “additive” rather than simultaneous in that not all features of the disease present at once; rather, they may present sequentially, requiring a high index of suspicion. While ESR and CRP can be mildly elevated in DM, as in our patient's case, they are often normal in the absence of lung or significant joint disease.

In addition to her joint and skin findings, the patient began to realize that she was parking her car closer to the front door of her workplace. She noted that she was becoming more breathless with any activity including walking and felt increasingly debilitated.

**Dermatology:** Although increased fatigue and breathlessness can be the result of deconditioning in patients with DM, we highly suspect these symptoms are due to ILD in this patient given her cutaneous findings described above. Once a patient with ILD in DM begins to experience pulmonary symptoms, their lung disease is quite advanced. Therefore, her history of increasing breathlessness should raise red flags for advanced lung disease. Prompt evaluation, workup, and treatment of her lung disease becomes increasingly crucial in her case.

**Rheumatology:** As mentioned, the fact that this patient is aware of her pulmonary symptoms suggests that ILD may already be present and potentially advanced. Her symptoms are likely due to interstitial changes in the lung parenchyma; however, breathlessness can also be a function of diaphragmatic weakness. When there is little or no systemic weakness, isolated diaphragmatic weakness is rare. Work-up here would include a high-resolution chest computerized tomography (CT) scan. In addition, we would obtain pulmonary function tests (PFTs) with diffusing capacity for carbon monoxide (DLCO) to determine the extent of restrictive lung disease and gas exchange compromise.

Her past medical history was unremarkable except for atopic dermatitis as a child. She denied taking any medication except for occasional ibuprofen when her joint pain became more severe.

**Dermatology:** DM is often mistaken for atopic dermatitis early in a patient's disease course. Clues to help differentiate the two include the distribution and color of skin lesions. In addition to Gottron's papules and proximal nailfold abnormalities, other pathognomonic cutaneous features of DM are:

• **V-sign:** erythema across the V-neck region of the chest
• **Shawl sign:** erythema or poikiloderma across the upper back
• **Heliotrope rash:** violaceous or erythematous hue of the bilateral upper eyelids, often associated with edema
• **Gottron's sign:** Pink to violaceous patches, papules, and/or plaques over the elbows and/or knees
• **Holster sign:** erythematous to violaceous macules and/or patches, often with livedoid or reticular changes, over the lateral thighs
• Other common cutaneous features seen in DM include photosensitivity, scalp erythema and/or dysesthesia, and midfacial erythema involving the nasolabial folds (contrary to the malar rash of acute cutaneous lupus erythematosus which spares nasolabial folds)

**Rheumatology:** Clues that this patient's arthritis is inflammatory in nature and therefore DM-related include its association with at least 30 minutes of morning stiffness, as previously noted, and improvement with ibuprofen.

Her family history was significant for several maternal relatives having **“thyroid problems.”**

**Rheumatology:** In my clinical experience, autoimmune thyroid disease, most often Hashimoto's thyroiditis (but also Graves’ disease), is often seen in combination in patients with myositis. While patients often have a family history of other autoimmune diseases, including autoimmune thyroid disease, myositis itself is rarely familial. Furthermore, there is a clear gender bias and women in particular are more susceptible to autoimmune disorders due to 1) hormonal changes that stimulate pro-inflammatory cytokine production and 2) genetic variability (the X chromosome has many more genes than the Y chromosome) causing a rise in polymorphic antigens and therefore an immune response (Angum et al., 2020).

On social history, she reported spending most of her day outdoors without sunscreen as an athletic coach. She reported drinking a moderate amount of alcohol, more often on weekends, and denied use of recreational substances or supplements.

**Dermatology:** Patients with DM are incredibly photosensitive. In fact, ultraviolet radiation has been shown to preferentially result in development of dermatomyositis in women, but not in men (Love et al., 2009). Therefore, counseling about strict photoprotection with both broad-spectrum physical sunscreen and photoprotective clothing for even brief sun exposure is key, particularly for our female patients. Alcohol history is important when considering treatment options, particularly if one is considering a medication such as methotrexate (MTX) with potential for hepatotoxicity. In addition to substances, patients should be asked about nutritional supplements, particularly green smoothies and juices, since previous research has postulated that certain herbal supplements including spirulina may play a role in DM onset (Bax et al., 2021, Lee and Werth, 2004).

Although we classically counsel patients about the risk of photosensitivity, some patients with DM, including those with anti-MDA5 phenotype, can also have a marked vasculopathic pattern to their disease with their cutaneous eruption flaring in the cold.

**Rheumatology:** Sun exposure exacerbates not only cutaneous features of DM but may also worsen muscle symptoms. In addition to being a potential contraindication to use of MTX, alcohol itself can be a myotoxin. Specifically, alcohol may be directly toxic to the muscle cells, causing muscle damage that may be hard to distinguish from underlying myositis.

Physical examination revealed a tall, thin woman in no acute distress. Her cutaneous exam is shown in Figs. 1-2. Her lungs had **diffuse, dry crackles most notable in the bases.** Musculoskeletal examination was notable for **synovitis across the MCP and PIP joints.** The strength in her neck flexors as well as proximal and distal upper and lower extremities was normal.

**Fig. 5:**
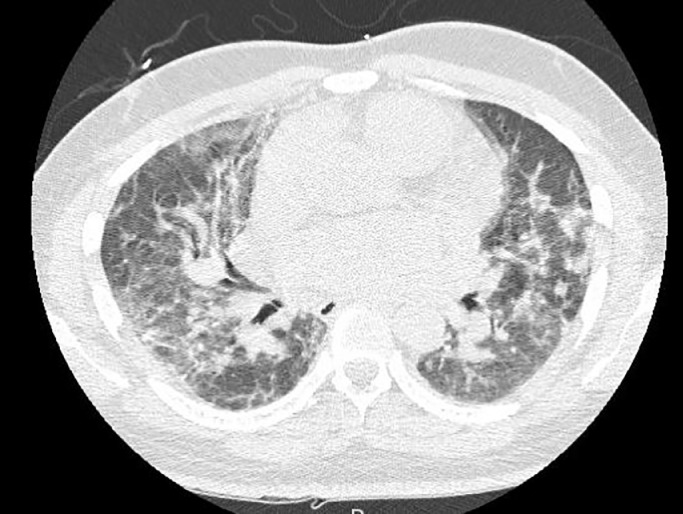
Computed tomography scan of the chest consistent with severe interstitial lung disease.

Her antinuclear antibody (ANA) and muscle enzymes (creatinine kinase [CK], aldolase, and lactate dehydrogenase [LDH]) were normal. Magnetic resonance imaging (MRI) of her bilateral thighs with T1 and STIR (fat suppressed) sequences was normal. Her chest CT was consistent with severe ILD (Fig. 5). PFTs revealed a diminished total lung capacity and reduced DLCO consistent with restrictive lung disease and impaired gas exchange.

**Dermatology:** Erythema in many inflammatory dermatoses may be more subtle in patients with skin of color. Hence, having a high index of suspicion for patients with darker skin tones who present with more subtle erythema, dyschromia/hyperpigmentation, and/or poikilodermatous change overlying the pathognomonic anatomic locations involved in DM is crucial to prevent diagnostic and treatment delays. Although DM is by and large a clinical diagnosis, a biopsy can be performed when there is diagnostic uncertainty. Biopsy of Gottron's papules or Gottron's sign classically shows interface dermatosis, whereas biopsy of the palmar papules in anti-MDA5 DM shows vasculopathy (Cao et al., 2012). Biopsy of the V-sign or shawl sign is more likely to be nonspecific.

At this point we are confident that this patient has anti-MDA5 DM, given her cutaneous features and advanced pulmonary disease. Although a myositis antibody panel was sent and eventually revealed a positive anti-MDA5 antibody, we do not rely on this panel for diagnosis or treatment. Importantly, the reliability of the myositis panel can vary by laboratory. For this reason, both of our institutions send our myositis panels to the Oklahoma Medical Research Foundation which specializes in myositis testing. Additionally, not all autoantibodies implicated in DM are included in a classic myositis antibody panel. For this reason, it is important to review what labs are included in any given myositis antibody panel and to add additional autoantibodies as needed.

**Rheumatology:** For this patient with progressive debilitation, we want to tease out the source of her fatigue and exercise intolerance. It could be secondary to her lung disease, or there may be early subtle proximal weakness that she has not noticed. Additionally, as patients develop more lung disease, they may be moving less often and therefore not be aware of skeletal muscle involvement. On our examination, we perform maneuvers to try to bring out subtle weakness which includes asking the patent to rise from a 6-inch stool, something that is difficult to do even with modest proximal hip flexor involvement. In cases where the clinical exam and labs are indeterminate, additional testing may also include an electromyogram (EMG) to assess for evidence of an irritable myopathy and a noncontrast MRI of the thighs. The thighs are the most common muscles that we image, as both thighs can be seen in one image, allowing for assessment of the symmetric nature of DM. Additionally, the thighs are frequently involved in classic DM, even if the patient has not fully recognized weakness in those muscles. We ask for T1 and STIR (fat suppressed) sequences to assess for abnormal muscle architecture (T1) as well as edema (STIR), which is often an indication of inflammation. MRI can also be very helpful for noting fasciitis which appears as a rim around key muscle groups such as the quadriceps or hamstrings. We can see isolated fasciitis in the absence of muscle edema in amyopathic DM. Finally, should we decide that a muscle biopsy is necessary, MRI can help localize the site for biopsy given that muscle involvement in DM is often patchy. Muscle biopsy is actually falling out of favor as the cutaneous features of DM are diagnostic, and myositis-specific antibodies can confirm the specific DM phenotype, which is initially suspected based on exam alone. Notably, for this patient, her muscle exam did not show any evidence of proximal muscle weakness, her muscle enzymes were normal, and MRI was unremarkable. As a result, her “weakness” is likely secondary to lung disease, underscoring the point that patients may describe weakness but are actually describing asthenia/fatigue due to their systemic illness. This is fitting for her case as anti-MDA5 DM is often amyopathic. Importantly, muscle disease can lag behind cutaneous findings. Therefore, patients without muscle disease are considered provisionally amyopathic after 6 months and confirmed amyopathic at 2 years (Sontheimer, 2002).

### Additional work-up and treatment

**Rheumatology:** Clinically amyopathic DM (CADM) has been found to have a significantly lower risk of cancer than other idiopathic inflammatory myopathies (Oldroyd et al., 2021). Furthermore, ILD is often thought to be protective against malignancy, so patients seem to have either ILD or cancer, but very rarely both (Chang et al., 2020). Nevertheless, cancer screening is still important in all patients with DM.

While MTX and azathioprine are often first-line therapies for DM, mycophenolate (MMF) is increasingly recognized as a first-line agent and is indicated for this patient's skin and lung disease. Systemic steroids should also be used given the pulmonary component. Severe pulmonary involvement is often treated with intravenous steroids initially. MMF is a weaker agent for arthritis and often not adequate for patients with prominent joint disease. Systemic steroids will likely be effective for her arthritis in the short term, with hydroxychloroquine added as a steroid-sparing adjunct for joint disease. Rituximab and/or intravenous immunoglobulin (IVIG) are often added for both refractory muscle and/or skin disease.

**Dermatology:** In addition to MMF for this patient's skin and lung disease, we often add anti-vasculopathic agents to help with ulceration and reduce morbidity in anti-MDA5 DM. The pathophysiology of anti-MDA5 DM is partially thought to be driven by an underlying vasculopathy, as described above in the biopsy findings of these patients. Vasodilators such as nifedipine and sildenafil help to alleviate underlying vasculopathy whereas aspirin and pentoxifylline help improve peripheral circulation.

While hydroxychloroquine (HCQ) is commonly used as an adjunctive agent in DM, it is not a particularly strong agent for the skin. In fact, most patients have no response to HCQ monotherapy (Pinard et al., 2019), and HCQ can lead to a cutaneous drug eruption, such as a morbilliform drug eruption, in almost a third of patients (Pinal-Fernandez et al., 2017, Pelle and Callen, 2002). Furthermore, some autoantibodies (anti-TIF-1γ, Mi-2, anti-NXP-2, anti-SAE-1/2, anti-MDA-5, anti-Jo-1, and anti-Ku) confer a higher risk of cutaneous adverse reactions to HCQ (Wolstencroft et al., 2018). From a dermatologic perspective, in DM, HCQ is most useful for its photoprotective benefits.

### Patient 2

A 63-year-old man presented for progressive **weakness and myalgias of his upper arms, thighs, lower arms, and legs for several weeks**. When questioned further, he reported paying little attention to his symptoms until he also started having **difficulty swallowing**. He also reported a new rash but had given it even less thought as it was subtler than this muscle weakness and pain.

**Rheumatology:** We are classically taught that DM manifests with painless proximal weakness. However, approximately 50% of patients with DM have muscle pain in addition to weakness. In DM, patients can have painless weakness or pain with weakness, but not pain without weakness (Tugwell et al., 2007). It is not uncommon for patients to have very significant muscle weakness before seeking medical attention. Here, this patient was not even aware of how weak he had become. Patients will often compensate for their weakness and attribute it to many other things (getting older, etc.) initially.

We are also classically taught that DM weakness is limited to the proximal extremities. In this case, the patient has both proximal and distal weakness, which is suspicious for the phenotype of DM associated with the anti-NXP2 autoantibody. This patient group is also more likely to have dysphagia (62% versus 35%; P < 0.001) (Albayda et al., 2017, Rogers et al., 2017). Dysphagia should be considered an emergency for patients with DM, requiring prompt attention and rapid treatment from the start, often requiring hospitalization.

**Fig. 6:**
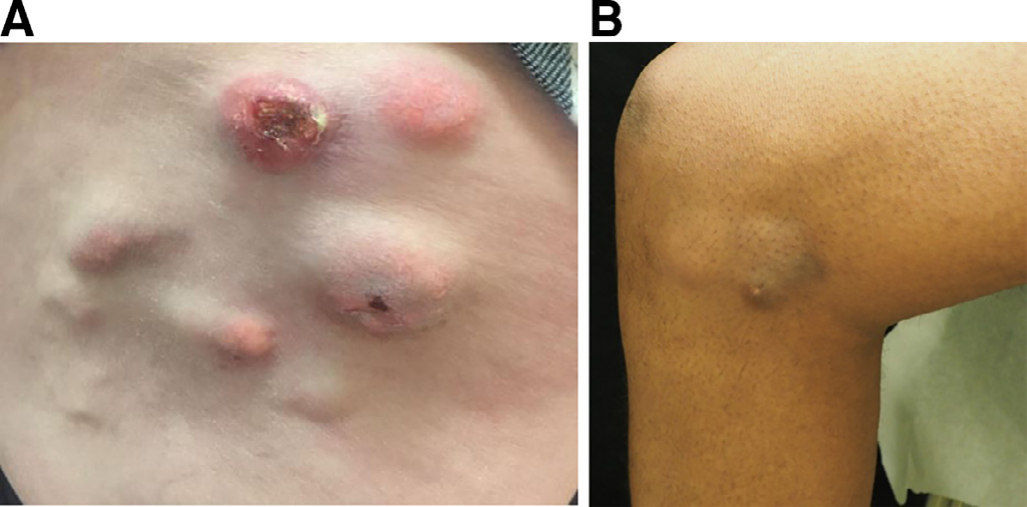
Firm, subcutaneous nodules within sites of severe muscle disease consistent with calcinosis cutis in a White patient (A) and patient with skin of color (B).

On physical examination, he had **mild periungual erythema and mildly violaceous papules and plaques on his forehead, elbows, knees, and V-neck region**. He had a subtle **violaceous hue over both lateral thigh** areas. In addition, there were **firm, subcutaneous nodules over the thighs, legs, and forearms, some with chalky white drainage (**Fig. 6**).**

His musculoskeletal examination revealed **proximal weakness** in the deltoids and hip flexors and neck flexors. He was **unable to lift his head** off of the examining table. Subtle **weakness of the wrist flexors and finger flexors** was also noted. He had no evidence of synovitis, and he had full range of motion in all joints tested.

**Dermatology:** While this patient has cutaneous features diagnostic of DM including periungual erythema, Gottron's sign, V-neck erythema, and holster sign, they are much more subtle than in Patient 1. What is more striking are the significant firm subcutaneous nodules over his thighs and forearms, consistent with calcinosis.

Calcinosis cutis can be seen in a number of connective tissue diseases secondary to calcium deposition at sites of inflammation or trauma. In DM, severe calcinosis is most seen in patients with severe muscle disease. As such, calcinosis can be a prominent complication in patients with juvenile DM, which almost invariably presents with muscle disease, as well as in adult patients with more severe muscle phenotypes such as anti-NXP2 DM. The subtle rash in this case further supports the NXP2 phenotype.

**Rheumatology:** As stated above, dysphagia is this patient's most concerning sign of severe and significant muscle disease. Physical exam findings further supporting the severity of his muscle disease are proximal muscle weakness, distal muscle weakness, inability to lift his head off the exam table, and calcinosis.

**Fig. 7:**
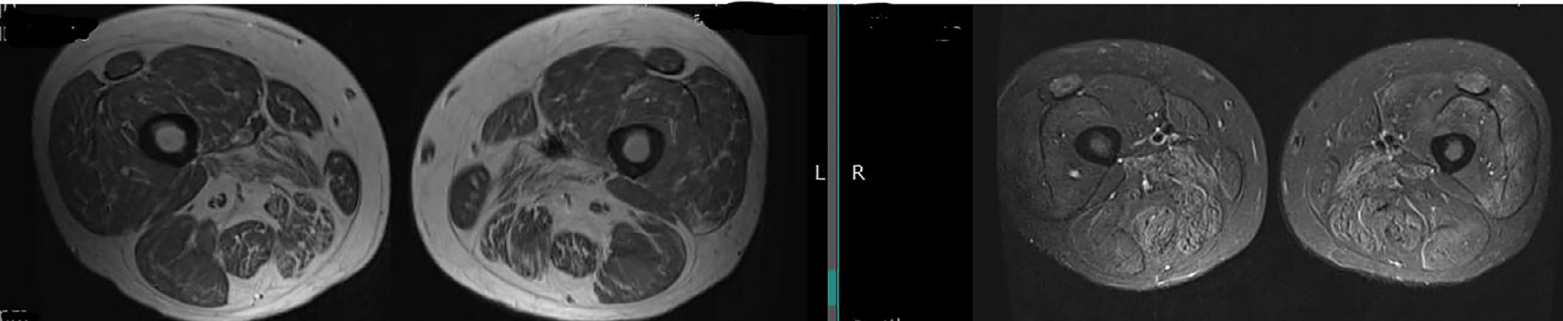
Magnetic resonance imaging of the bilateral thighs showing fatty replacement on T1 sequence (left) and moderate edema on STIR (fat suppressed) sequence (right), consistent with myositis.

His laboratory testing noted a normal **CK of 48 units/L**, mildly elevated **aldolase of 9.0 units/L, slightly elevated aspartate aminotransferase (AST) of 35 units/L,** and normal ESR and CRP. Complete blood count and comprehensive metabolic panel were otherwise normal. An MRI of the bilateral thighs revealed **diffuse edema-like signal** in most muscles (Fig. 7). An EMG showed an **irritable myopathy**. An ANA was positive at 1:160 speckled, and a myositis panel ultimately revealed an anti-NXP2 antibody.

**Rheumatology:** CK may be normal in the face of severe muscle disease in DM. DM is often associated with substantial inflammation but little muscle necrosis, which may explain why the CK leak is minimal. Aldolase may be high since it may rise before CK rises or it can be secondary to fascial involvement, which is a prominent feature in DM (Casciola-Rosen et al., 2012). EMG testing can be important to determine evidence for an irritable myopathy. An MRI of the thighs helps quantify fascial edema and muscle edema. We must be extremely cautious of calling DM amyopathic merely on the basis of a normal CK. Importantly, patients with skin of color with myositis often have worse clinical outcomes (Casciola-Rosen et al., 2012). Thus, prompt recognition of myositis, especially in patients with skin of color, is of paramount importance.

A quick note about the ANA: 4 of the 5 myositis-specific antibodies associated with DM (Mi-2, anti-TIF-1γ, anti-SAE1/2, anti-NXP2) target nuclear antigens and are therefore associated with a positive ANA. Other myositis-specific antibodies, such as anti-MDA5, are cytoplasmic; thus, these patients will often have a negative ANA. As in all cases of DM, the myositis antibody panel helps confirm a clinical phenotype based on history, exam, and imaging. Although this patient's anti-NXP2 antibody was ultimately confirmed, this case serves as another reminder that clinical diagnosis and treatment must be established without relying on autoantibody results to guide initial therapies.

On further history, he complained of diffuse abdominal pain and recent **unintentional weight loss of 20 pounds**. CT scan showed **a renal mass.** A PET scan revealed a hypermetabolic area at the mass noted in the left upper renal pole. He underwent a partial nephrectomy which showed early renal cell carcinoma. A colonoscopy was unremarkable.

**Rheumatology:** Compared to other idiopathic inflammatory myopathies, DM is significantly associated with a higher risk of cancer (Oldroyd et al., 2021). Risk factors that increase cancer risk include older age, male sex, antibodies to NXP-2 or TIF-1γ [17], and dysphagia (Oldroyd et al., 2021). Recent meta-analyses have not shown the NXP-2 association with cancer to be robust, however, in comparison to other myositis autoantibodies such as anti-TIF-1γ (Oldroyd et al., 2021).

Given that we know this patient has cancer, his DM is most likely paraneoplastic to his underlying malignancy. There is no consensus on malignancy screening; a recent systematic review determined that CT scanning could be useful but concluded that further prospective studies and guidelines are needed (Oldroyd et al., 2021).

Although ILD and malignancy are rarely found in combination, this patient should still have a CT scan of his chest, as early ILD may be minimally symptomatic. An initial CT scan is done to document any subtle ILD even when PFTs may still be normal. If PFTs are abnormal, then we may repeat a CT scan. In addition to ILD, childhood onset and arthralgias may be protective factors for cancer in patients with myositis (Liu et al., 2018).

**Dermatology:** In the absence of established malignancy screening guidelines, at our institution, we screen annually for the first 3 years after DM onset with age- and sex-appropriate tests (colonoscopy, mammogram, Pap smear), CT of chest, abdomen, pelvis, transvaginal pelvic ultrasound, and occasionally tumor markers such as a CA-125 given the higher association with ovarian cancer. For women, the most frequent myositis-associated malignancies include ovarian and breast cancer and is therefore important to screen for these malignancies for our female patients (Hsu et al., 2021).

### Additional work-up and treatment

**Dermatology:** This patient warrants aggressive treatment of his severe muscle disease due to the risks associated with dysphagia as well as his calcinosis, which is the end result of his muscle disease.

The most crucial aspect of this patient's treatment is to control his muscle disease rapidly. In patients with severe muscle disease, MTX and IVIG are often used concomitantly as first-line steroid-sparing agents. The primary role of IVIG is to help gain rapid control of muscle disease, which is the cause of his progressive calcinosis. However, there are also some data supporting the use of IVIG for calcinosis itself (Hoeltzel et al., 2014). Although medical management of calcinosis is notoriously difficult, potential adjunctive treatments that can be considered include high-dose diltiazem, colchicine, and intravenous and/or intralesional sodium thiosulfate. Systemic steroids are crucial for severe muscle disease while steroid-sparing agents take effect but are not consistently effective for the cutaneous manifestations of DM and thus not first-line from a dermatologic perspective.

**Rheumatology:** Treatment for patients with dysphagia often includes high-dose systemic steroids, IVIG, and an additional disease-modifying antirheumatic drug like methotrexate (MTX). The goal is to treat quickly to try to avoid a percutaneous endoscopic gastrostomy tube. In this case, MTX is favored as it is compatible with the patient's underlying malignancy. This patient is a male, so we need not have to take into consideration its teratogenic effects. However, when coming up with a management plan, it is important to ask our female patients who are in childbearing years about their thoughts around family planning. Unlike MTX and MMF, IVIG is safe in pregnancy and breast feeding.

In this patient, however, MTX is not contraindicated due to this patient's elevated liver function tests, which likely stem from myositis rather than liver inflammation. Checking a gamma-glutamyl transferase can help discern when transaminases are of liver, rather than of muscle, origin. Work-up for patients with dysphagia in DM often includes a cine esophagram to determine severity and quantify aspiration risk. An interdisciplinary approach incorporating speech therapy is also important. Tumor removal may be associated with DM remission, but this is not always the case, unfortunately.

## Conclusion

These two distinct cases of dermatomyositis highlight how patients with DM benefit from a comprehensive collaborative approach to their diagnosis and management. As you can see, DM is an extremely complex disease and it is of no surprise that it has shown to have a significant impact on quality of life when compared to various other dermatologic and non-dermatologic diseases (Goreshi et al., 2011). Unfortunately, DM affects more of our female population and it is important for physicians of all disciplines to include psychosocial interventions for our patients with DM.
